# Robust and Accurate Mandible Segmentation on Dental CBCT Scans Affected by Metal Artifacts Using a Prior Shape Model

**DOI:** 10.3390/jpm11050364

**Published:** 2021-05-01

**Authors:** Bingjiang Qiu, Hylke van der Wel, Joep Kraeima, Haye Hendrik Glas, Jiapan Guo, Ronald J. H. Borra, Max Johannes Hendrikus Witjes, Peter M. A. van Ooijen

**Affiliations:** 1 3D Lab, University Medical Center Groningen, University of Groningen, Hanzeplein 1, 9713 GZ Groningen, The Netherlands; b.qiu@umcg.nl (B.Q.); h.van.der.wel@umcg.nl (H.v.d.W.); h.h.glas@umcg.nl (H.H.G.); m.j.h.witjes@umcg.nl (M.J.H.W.); 2 Department of Radiation Oncology, University Medical Center Groningen, University of Groningen, Hanzeplein 1, 9713 GZ Groningen, The Netherlands; j.guo@umcg.nl (J.G.); p.m.a.van.ooijen@umcg.nl (P.M.A.v.O.); 3 Data Science Center in Health (DASH), University Medical Center Groningen, University of Groningen, Hanzeplein 1, 9713 GZ Groningen, The Netherlands; 4 Department of Oral and Maxillofacial Surgery, University Medical Center Groningen, University of Groningen, Hanzeplein 1, 9713 GZ Groningen, The Netherlands; 5 Medical Imaging Center (MIC), University Medical Center Groningen, University of Groningen, Hanzeplein 1, 9713 GZ Groningen, The Netherlands; r.j.h.borra@umcg.nl

**Keywords:** accurate mandible segmentation, oral and maxillofacial surgery, 3D virtual surgical planning (3D VSP), convolutional neural network

## Abstract

Accurate mandible segmentation is significant in the field of maxillofacial surgery to guide clinical diagnosis and treatment and develop appropriate surgical plans. In particular, cone-beam computed tomography (CBCT) images with metal parts, such as those used in oral and maxillofacial surgery (OMFS), often have susceptibilities when metal artifacts are present such as weak and blurred boundaries caused by a high-attenuation material and a low radiation dose in image acquisition. To overcome this problem, this paper proposes a novel deep learning-based approach (SASeg) for automated mandible segmentation that perceives overall mandible anatomical knowledge. SASeg utilizes a prior shape feature extractor (PSFE) module based on a mean mandible shape, and recurrent connections maintain the continuity structure of the mandible. The effectiveness of the proposed network is substantiated on a dental CBCT dataset from orthodontic treatment containing 59 patients. The experiments show that the proposed SASeg can be easily used to improve the prediction accuracy in a dental CBCT dataset corrupted by metal artifacts. In addition, the experimental results on the PDDCA dataset demonstrate that, compared with the state-of-the-art mandible segmentation models, our proposed SASeg can achieve better segmentation performance.

## 1. Introduction

**Figure 1:**
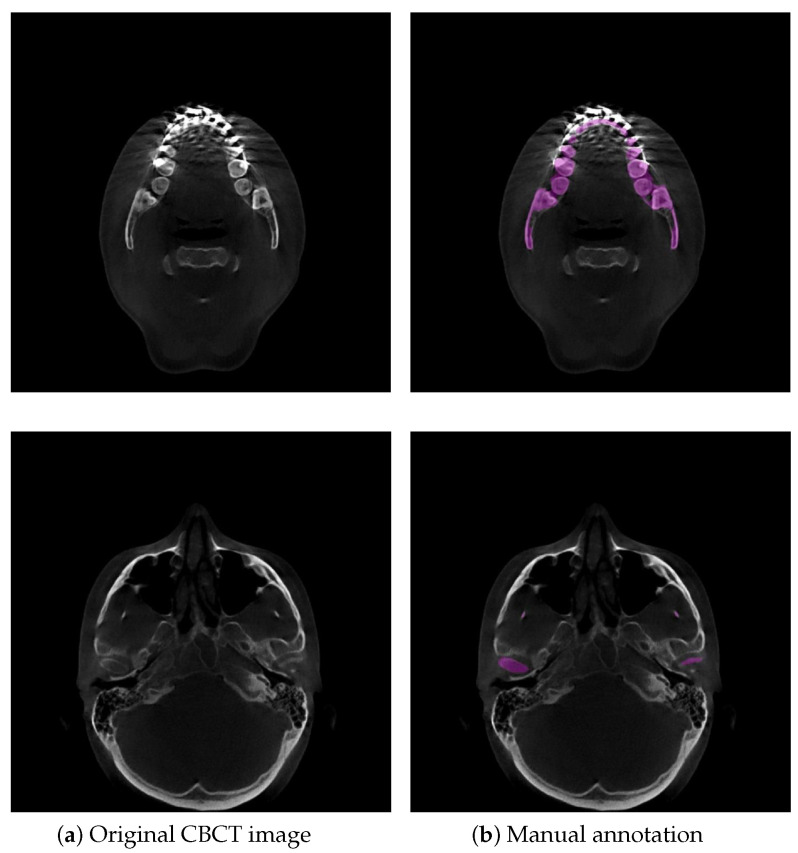
Example illustrations that challenge mandible segmentation in CBCT images. (**a**) Original CBCT image. The mandible and teeth appear with almost invisible boundaries. (**b**) Example of manual annotation. Low contrast often appears in condyles. The purple region indicates the manual annotation of the mandible.

Currently, the three-dimensional (3D) virtual surgical planning (VSP) technique is commonly used for oral and maxillofacial surgery (OMFS), and planning since it allows for pre- or postoperative simulation of surgical options [1]. 3D surface models of the mandible in 3D VSP are created and superimposed to visually and quantitatively demonstrate the orthodontic/orthognathic changes and provide postoperative follow-up of patients with cranio-maxillofacial deformities [1]. Cone-beam computed tomography (CBCT) is widely applied in 3D VSP because of its lower radiation dose and faster scanning time than conventional CT [2]. In orthodontic or orthognathic treatment, the dentist or maxillofacial surgeon needs visual information about the location and movement of their patient’s teeth and mandible. A requirement for this process is to accurately segment the mandible from the dental CBCT scans and then to generate 3D surface mandible model. Therefore, accurate mandible segmentation plays an important role in 3D VSP for OMFS. Dental CBCT scans are noisier and have more metal artifacts than conventional CTs because dental CBCTs use a low-radiation technique and teeth, dental braces in orthodontic treatment and metal implants in orthognathic treatment are higher attenuation materials, easily leading to high noise and strong metal artifacts in the visual impression of the scans [3]. The boundaries of mandibles are difficult to be identified since dental braces and metal implants badly affect the image quality in CBCT [4], as shown in Figure 1. Additionally, low contrast in the condylar process very often leads to ambiguous and blurred boundaries in CBCT scans because of its low radiation dose, as illustrated in Figure 1. Consequently, the main difficulty in orthodontics or orthognathic visualization is precise mandible segmentation in CBCT scans. Currently, manual segmentation for 3D modeling of the mandible is widely adopted in clinical practice, but this is a time-consuming and labor-intensive approach so that it is impractical to perform on a large number of subjects. Moreover, manual segmentation often suffers from large interoperator variability (Dice score of 94.09% between two clinical experts) [5], which directly influences the quality of treatment planning. To date, there are still no reliable automatic segmentation approaches that can adapt to badly affected CBCT scans. Accordingly, it is meaningful to develop an accurate and automatic technique to segment the mandible for orthodontic or orthognathic treatment from CBCT images.

To reduce the workload of mandible segmentation, a number of (semi)automatic segmentation methods on CBCT have been developed. Wand et al. [6] proposed an automated segmentation method from dental CBCT images using patch-based sparse representation and convex optimization. They used the B-spline registration algorithm provided by the Elastix toolbox for the deformable registration. Furthermore, the average computational time was approximately 5 h for segmentation of a scan of size 400×400×400 [6]. Fan et al. [7] used a marker-based watershed transform method for fully automatic mandible segmentation from CBCT images. This approach used a Gaussian filter and manually selected an adequate threshold for preprocessing initialization. Gollmer et al. [8] employed a statistical shape model (SSM) with optimized correspondence, which can help to improve the segmentation accuracy of the mandible. This method needs to manually and intentionally choose the proper mandible prior as initialization and a suitable window width of the segmentation object. Oscar et al. [9] proposed an interactive segmentation method to aid specialists in segmenting CBCT via applying supervoxels and graph clustering techniques. Wang et al. [10] presented a majority voting method and combined it with a random forest for mandible segmentation in dental CBCT. These approaches have been proven to be useful in utilizing prior knowledge in the segmentation tasks [11]. However, these methods suffer from the problem of hyperparameter selection and the manual positioning of landmarks in the initialization steps [12]. The performance of the methods is often affected by high noise or metal artifacts caused by dental braces or implants [13,14]. In other words, the application of these traditional techniques requires expert analysis and adjustment in every specific patient, which makes it difficult to handle the massive amount of medical data encountered in practice.

**Figure 2:**
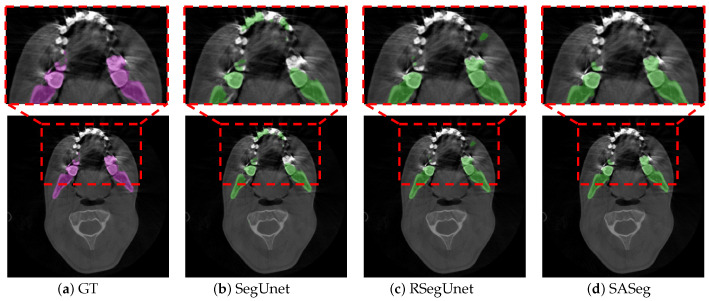
An example illustration that shows the comparison with SegUnet and RSegUnet. The existing state-of-the-art convolutional neural network (CNN) approaches for segmentation tasks perform poorly when the input data are strongly degenerated by noise. (**a**) the ground truth segmentation; (**b**–**d**) the automatic segmentation results obtained from SegUnet [27], RSegUnet [26] and the proposed SASeg. The purple region indicates the manual annotation, while the green regions indicate automatic segmentations.

With the development of deep learning technology, deep learning methods have been proven to show powerful capabilities in detailed image feature extraction in automatic segmentation tasks [15,16,17]. The deep learning approach provides much more flexibility than the traditional methods [6,7,8,9,10], which require less expert analysis and fine-tuning and can easily exploit the other objects [18]. However, these studies [15,16,17] are still not robust in segmenting organs because the medical image usually has a 3D volume form, but 2D slices are usually fed as the input. For instance, Minnema et al. [13] employed a mixed-scale dense convolutional neural network to segment the mandible in CBCT. However, the other bone-structured organs are also segmented in this network, as shown in the figures of [13], due to the fact that the spatial information was not considered in their 2D network. To use 3D spatial information from the volume data, researchers first proposed to use a 3D network instead of the original 2D network. Çiçek et al. [19], Milletari et al. [20], Zhu et al. [21], and Wang et al. [22] explored a 3D convolution kernel in their network instead of the original 2D kernel in the medical image segmentation task. However, fully implementing the 3D network for image segmentation requires cropping the input volume into a small patch in training, which limits the maximum receptive field of the network and leads to the loss of global information. It is difficult for the 3D network to learn the overall structure information of the target. Thus, researchers started to investigate learning 3D spatial information and voxel connectivity of the upper and lower context of the object via a 2D network. Mortazi et al. [23] proposed the multiplanar training strategy, which utilized the images’ three perpendicular planes (axial, coronal, and sagittal) as input. Novikov et al. [24] used a sequence of slices as input in the network. Qiu et al. [12] adopted a 2.5D volume as input in their network and then combined the resulting 2D segmentations from three orthogonal planes into a 3D segmentation. Ghavami et al. [25] incorporated different numbers of neighboring slices as input for prostate segmentation from ultrasound images. Qiu et al. [26] developed a novel technique that combined a regular segmentation network with a recurrent module in their network for mandible segmentation in conventional CT scans. In general, these works [12,23,24,25,26] have shown that using adjacent slices can help the network obtain more accurate and reliable results in terms of anatomy. Nevertheless, these works [12,23,24,25,26] have also shown that obtaining 3D spatial information from a 2D network still leaves room for improvement. As illustrated in Figure 2, an example shows the comparison between SegUnet [27], recurrent SegUnet (RSegUnet) [26] and the proposed method in mandible segmentation. These methods are not suitable for CBCT images that are strongly corrupted by the metal braces and low contract due to the inherent characteristic of CBCT.

With the development of conventional approaches and the deep segmentation neural network, introducing prior information to the network has become a popular topic in research. Chen et al. [28] tried to use a shape prior in their segmentation model. They first used the deep Boltzmann machine to extract the prior shape hierarchy, which can capture global and local structures of the prior shapes. Then, the learned structure is introduced in an energetic form to regularize the target shape for image segmentation [28]. Duan et al. [29] proposed a context-aware 3D fully convolutional network (FCN) for vessel enhancement and segmentation in coronary computed tomography angiography volume, which used 3D vessel-like structures as spatial prior knowledge to feed the 3D FCN. Tong et al. [30] developed a novel automated segmentation approach incorporating shape priors as a constraint term, where they combined an FCN with a shape representation model. However, how to define an appropriate prior shape model to guide object segmentation is still an open problem.

Motivated by the above analysis, we propose a novel convolutional neural network (CNN) framework based on shape-aware segmentation for mandible segmentation (SASeg), which follows the classical encoder–decoder structure. We first adopt a prior shape feature extractor (PSFE) to capture the shape information from the mean mandible shape model, in which the mean model is implemented in the average mandible shapes in the training set. In this way, the network can be aware of the general mandible shape information. To avoid the large computation and memory demand in the 3D network and utilize spatial information from the 3D volume, we feed the mean mandible shape feature from PSFE into a recurrent FCN for mandible segmentation at the pixel level.

In particular, our main contributions are as follows: (1) We present a novel end-to-end method for dental CBCT mandible segmentation based on prior shape information. (2) We propose a PSFE module to extract the spatial mandible information from the mean mandible shape model. (3) The proposed method provides the potential capability of removing the need for post-processing steps, even in cases where the images are corrupted by metal artifacts or noise due to the limitations in their image acquisition.

## 2. Methodology

**Figure 3:**
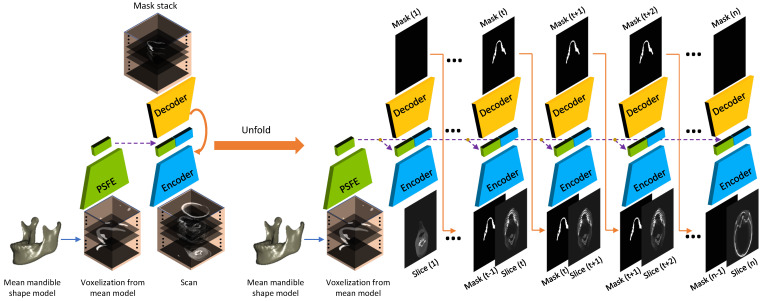
Overview of the proposed SASeg and its corresponding unfolded computational graph. The PSFE module is leveraged to extract general shape features from a mean mandible shape, and recurrent SegUnet connections are used to conduct the slice-by-slice segmentation.

In this section, we elaborate on the construction of the proposed SASeg and its core modules (i.e., PSFE, recurrent SegUnet). Figure 3 demonstrates the proposed SASeg, which builds recurrent SegUnet (RSegUnet) connections between adjacent units to retain the connectivity of mandible anatomy and utilizes the common mandible shape feature by extracting the mean mandible model from PSFE. Each unit in the RSegUnet is implemented as a classic end-to-end segmentation architecture for 2D slice segmentation. Moreover, every unit consists of an encoder and a decoder, as indicated by the blue and yellow ladder blocks in Figure 3. The PSFE module is inserted at the last layer of the encoder to capture mandible prior information, as indicated by the green ladder block in Figure 3.

Specifically, we first use the preprocessing technique of the statistical shape model (SSM) [31,32] to generate a mean mandible shape structure in the training set based on manual expert segmentations. In this way, every mandible shape is represented in the average mandible shape. The average mandible shape is then introduced into the proposed framework as a shape prior input in the network. To obtain more abundant structure knowledge, we employ a prior shape feature extractor (PSFE) as an auxiliary path to encode the average mandible shape so that the common features of the mandibles can be obtained. PSFE makes use of the mandible shape information from the mean model to help supervise the modeling of the target area, which is helpful to refine the segmentation performance. Furthermore, we employ the recurrent SegUnet in the framework, which has been proven helpful to segment 3D objects in 2D networks [26]. The proposed network that extracts prior knowledge from the mean shape model is able to constrain the mandible shape consistency in the segmentation task. Details of PSFE and the recurrent SegUnet in the proposed method are introduced as follows.

### 2.1. Building a Mean Mandible Shape Model

**Figure 4:**
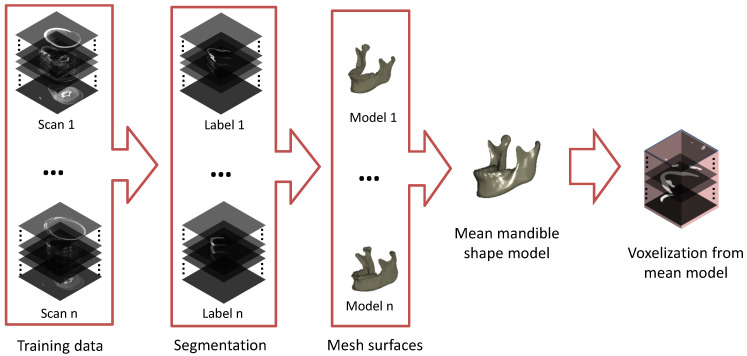
The procedure of generation of the mean mandible shape model. The generation consists of preparing the mandible model, generating the mesh surfaces, creating the mean mandible shape model, and calculating the corresponding mask stack.

We present a brief overview of the mean mandible shape model generation process, as illustrated in Figure 4. For generating a mean mandible shape model, we use a similar method for the preprocessing of SSM [31]. A dataset of mandible shapes is needed for generating a mean mandible shape model. The required mandible shapes are obtained via manual segmentation by an experienced technical physician and surface processing methods in the training set. The mandible shapes vary widely in terms of rotation, scale, and position of the object [33]. Therefore, all *n* mandibles need to be aligned into a common coordinate frame. This can be achieved by applying a generalized Procrustes alignment to remove all information that is unrelated to shape (i.e., rotation, position and scaling information) [34]. The mean mandible shape model is built from the set of *n* rigidly-aligned input shapes, which have been remeshed so the mandible shape is described by *l* landmark points which are forming a triangulation mesh over the contour of the mandible with edge lengths of ±1 mm. Using a surface registration method based on elastic deformation [35], correspondence between the landmark points on the input shapes was established. Due to the registration steps, the variation in position of the corresponding landmarks on the input mandibles now represents the shape variation for each landmark. Based on this, the mean mandible shape was calculated. To adapt the mean mandible model to the network, we voxelize the mean 3D mandible geometry into the same image coordinate system. The data processing pipeline for generation of the mean mandible shape model fed to the network is presented in Figure 4. The main stages of the method are illustrated as follows: preparation of training data, manual segmentation, surface processing (i.e., Procrustes alignment), building a mean mandible model, and voxelization back to the image coordinate system. For convenience, we still use the mean mandible shape model to represent the voxelized mean mandible shape model.

### 2.2. Prior Shape Feature Extractor (PSFE)

**Figure 5:**
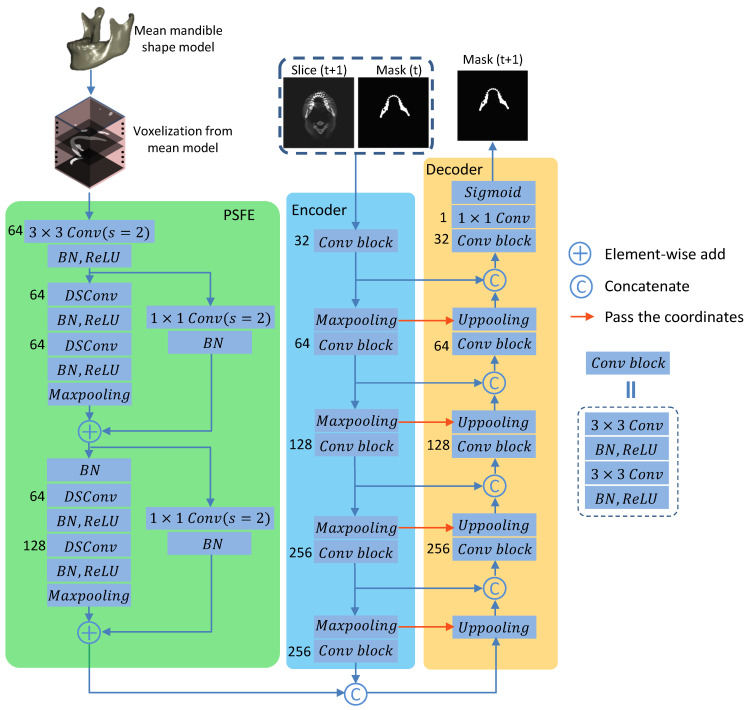
Details of each unit of SASeg. Each unit of SASeg consists of the PSFE, an encoder and a decoder. The number of channels is indicated in the left of each convolution. The PSFE module is inserted at the last layer of the encoder to capture the mandible prior information.

Using a mean shape model as the global contextual prior has proven to be a promising approach and is commonly used in conventional image segmentation tasks [36,37]. These works motivated our development of the prior shape feature extractor (PSFE) using the mean mandible shape model as a shape prior in the segmentation network. The PSFE architecture consists of one convolution block (a 3 × 3 convolution with a stride of 2, batch normalization (BN), ReLU) and two residual blocks (two depthwise separable convolutions (DSConv) [38], BN, ReLU, Maxpooling), as shown in Figure 5. To capture sufficiently large amount of contextual information, the feature maps are gradually downsampled in PSFE architectures. The mean mandible shape model is fed into the PSFE to learn mandible prior information to improve the performance of mandible segmentation with regard to complex situations such as blurred dental CBCTs, as illustrated in PSFE of Figure 5. The PSFE module is connected to the last bottom layer of the encoder, which is introduced in Section 2.3. The PSFE module learns $\theta_{PSFE}$ from the prior mandible information obtained from the mean mandible shape model. It can reduce oversegmentation (i.e., false positive prediction) in the segmentation task.

DSConv [38] in PSFE is improved from the Inception v3 structure [39]. DSConv makes the network processing simpler and more effective [38], especially in regard to increasing the computational efficiency. DSConv consists of depthwise convolutions and pointwise convolutions (i.e., 1×1 convolutions). Depthwise convolution first performs a 3×3 convolution operation for each input channel. Then, pointwise convolution performs 1×1 convolution to fuse the feature maps after the depthwise convolution. Assume that the number of channels in the *i*-th layer is $C_{i}$. The channels are independent of each other, so the number of parameters of the convolution kernel is $3\times 3\times C_{i}$, which is much less than $3\times 3\times C_{i}\times C_{i-1}$ of standard convolution. After channel-by-channel convolution, a 1×1 convolution kernel is used for feature fusion between channels. Therefore, the number of parameters of the second half of the convolution kernel is $1\times 1\times C_{i-1}\times C_{i}$. Furthermore, the calculation amount of the DSConv is $3\times 3\times C_{i-1}\times H\times W+1\times 1\times C_{i-1}\times C_{i}\times H\times W$, where *H* and *W* present the weight and height of the feature maps, respectively. The calculation amount of using DSConv is $\frac{1}{C_{i}}+\frac{1}{9}$ of the standard convolution, which is $3\times 3\times C_{i-1}\times C_{i}\times H\times W$. Therefore, the amount of calculation is greatly reduced. Moreover, PSFE does not require the training of multiple models and a large number of extra model parameters. Here, the number of trainable parameters of PSFE in SASeg is 149,440. Furthermore, using PSFE as an auxiliary path to extract the mean mandible shape information can be easily applied in a segmentation network with a small increase in the amount of memory and computational resources.

### 2.3. Recurrent Convolutional Neural Networks for Segmentation

We start from SegUnet [27] as the node in the recurrent architecture. Similar to U-Net [15], the basis network we use in SASeg consists of an encoder and a decoder path, each with four resolution steps. In the encoder path, each layer has two 3×3 convolutions, each followed by a batch normalization (BN) [40], a rectified linear unit (ReLU) [41] and a 2×2 max pooling with strides of two in each dimension. In the decoder path, each layer has an upsampling layer of 2×2 followed by two convolutions with a kernel size of 2×2, each followed by a BN and an ReLU as well. The resulting feature maps from each resolution in the encoder are concatenated to those of the same resolution in the decoder. In the last layer, a 1×1 convolution is used to reduce the number of output channels to the number of labels, which is 1 in a binary classification problem. The SegUnet network architecture is shown in the encoder and decoder in Figure 5. A recurrent convolutional neural network (CNN) algorithm is introduced to segment the mandible iteratively. At each iteration, the original input slice and previous probability map are fed into the network. This kind of network was robust for segmentation of the mandible from CT images due to learning the 3D spatial information and voxel connectivity of the upper and lower context of the object [26]. Different from the original encoding and decoding structure, we concatenate the feature maps including prior information in the bottom layer, which is obtained from a PSFE.

### 2.4. Combo Loss Function

Motivated by a combo loss used in [42], a combination of Dice and binary cross-entropy (BCE) loss have been applied to train the proposed SASeg. These loss functions are selected due to their potential to contend with imbalanced data [42].

(1) $$L=\omega_{1}\times L_{BCE}+\omega_{2}\times L_{Dice},$$

where $\omega_{1}$ and $\omega_{2}$ are the trade-offs between the BCE term and Dice term contribution in the loss L, which are set as $\omega_{1}=1$ and $\omega_{2}=1$ to obtain the gradient update. $L_{BCE}$ and $L_{Dice}$ are defined as follows:

(2) $$L_{BCE}\left(\hat{y},y\right)=-\frac{1}{N}\sum_{i=1}^{N}y_{i}\log\left({\hat{y}}_{i}\right)+\left(1-y_{i}\right)\log\left(1-{\hat{y}}_{i}\right),$$

(3) $$L_{Dice}\left(\hat{y},y\right)=1-\frac{2\sum_{i=1}^{N}y_{i}{\hat{y}}_{i}}{\sum_{i=1}^{N}y_{i}+{\hat{y}}_{i}}.$$

Here, $y_{i}$ and ${\hat{y}}_{i}$ represent the ground truth and the predicted probability of pixel *i*, respectively, and *N* is the number of pixels.

### 2.5. Dataset

A total of 59 dental CBCT scans that had been heavily affected by metal artifacts were used in this study. All the CBCT scans are obtained on a Vatech PaXZenith3D or Planmeca promax. Each scan consists of 431 to 944 slices with a size of 992×992 to 495×495 pixels. The pixel spacing varies from 0.2 to 0.4 mm, and the slice thickness varies from 0.2 to 0.4 mm. Of these CBCT scans, 38 are used for training, 1 is used for validation, and 20 are used for testing. To train a CNN for bone segmentation in these CBCT scans, gold standard segmentation labels were required. These gold standard labels were created by manually segmenting all CBCT scans using Mimics software (Mimics Medical 23.0, Materialise, Leuven, Belgium) by one experienced technical physician. Gold standard segmentations are actually segmentations augmented with a dentition model. This task took approximately 30–60 min per scan.

To investigate the generalization ability of our model, we also compare our proposed method with several state-of-the-art methods on a public CT dataset, which is the Public Domain Database for Computational Anatomy (PDDCA) version 1.4.1 used for the “Head and Neck Auto Segmentation MICCAI Challenge (2015)” [43]. There are 48 patient CT scans from the Radiation Therapy Oncology Group (RTOG) 0522 study in the PDDCA dataset, in which 40 out of the 48 patients in PDDCA with manual mandible annotations were used in previous studies [43,44]. According to the protocol setting in the PDDCA challenge description, 40 cases are separated into 25 cases (0522c0001-0522c0328) as a training subset and 15 cases (0522c0555-0522c0878) as a testing subset [43]. Each scan consists of 76 to 360 slices with a size of 512×512 pixels. The pixel spacing varies from 0.76 to 1.27 mm, and the slice thickness varies from 1.25 to 3.0 mm [43].

### 2.6. Evaluation Metrics

For quantitative analysis of the experimental results, four performance metrics are used, including the Dice coefficient (Dice), the average symmetric surface distance (ASD), and the 95% Hausdorff distance (95HD).

The Dice coefficient is widely used to assess the performance of image segmentation algorithms [45]. It is defined as:

(4) $$\mathrm{Dice}=\frac{2\sum_{i=1}^{N}y_{i}{\hat{y}}_{i}}{\sum_{i=1}^{N}y_{i}+{\hat{y}}_{i}},$$

where $y_{i},{\hat{y}}_{i}$ represents the ground truth and the predicted probability of pixel *i*, respectively, and *N* is the number of pixels.

The average symmetric surface distance (ASD) is a measure of computing the average distance between the boundaries of two object regions [30]. It is defined as:

(5) $$\mathrm{ASD}\left(A,B\right)=\frac{d(A,B)+d(B,A)}{2},$$

(6) $$d\left(A,B\right)=\frac{1}{N}\sum_{a\in A}\min_{b\in B}∥a-b∥,$$

where ∥.∥ represents the $L_{2}$ norm. *a* and *b* are corresponding points on the boundary of *A* and *B*.

The Hausdorff distance (HD) measures the maximum distance of a point in a set *A* to the nearest point in the other set *B* [46]. It is defined as:

(7) HD(A,B)=max(h(A,B),h(B,A))

(8) $$h\left(A,B\right)=\max_{a\in A}\min_{b\in B}∥a-b∥$$

where h(A,B) means the directed HD. The maximum HD is sensitive to contours. When the image is contaminated by noise or occluded, the original Hausdorff distance is prone to mismatch [43,47]. Thus, Huttenlocher proposed the concept of partial Hausdorff distance in 1933 [46]. The 95HD metric is similar to the maximum HD. In brief, 95HD selects 95% of the closest points in set *B* to the point in set *A* in Equation (8) to calculate h(A,B):

(9) $$95\mathrm{HD}=\max(h^{95\%}\left(A,B\right),h^{95\%}\left(B,A\right))$$

(10) $$h^{95\%}\left(A,B\right)=\max_{a\in A}\min_{b\in B^{95\%}}∥a-b∥$$

The purpose of using 95HD is to reduce the impact of a small subset of inaccurate prediction outliers on the overall assessment of segmentation quality.

### 2.7. Implementation Details

The proposed CNN model is implemented on an NVIDIA GeForce Tesla V100 or Quadro P6000 by using PyTorch 1.4.0 [48]. In this study, the batch size, epochs, and learning rate are set to 3, 50, and 0.0001, respectively. Furthermore, the Adam optimizer is used to minimize the combo loss. Finally, an early stopping strategy is applied if no improvement in the loss of the validation set for five epochs occurs to avoid overfitting. We apply n=39/25 mandible shapes in the training set to build mean mandible shape model for the CBCT/PDDCA dataset.

## 3. Results

### 3.1. Method Comparison

We compared the proposed SASeg with recent fashionable methods, such as U-Net [15], SegNet [16], SegUnet [27], and AttUnet [17], which are widely used in medical image segmentation. In order for fair comparison, the same parameter settings are applied in those methods. In Table 1, we list the quantitative results as well as the number of parameters of the corresponding approaches. As shown in Table 1, the proposed SASeg gets the best performance and achieves approximately 0.55% to 4%, 0.76 mm to 3 mm and 13.4 mm to 37 mm gains in terms of Dice, $D_{ASD}$ and $D_{95HD}$, respectively. Furthermore, the results in Table 1 show that our proposed SASeg is more effective in segmenting the mandible, though there is a small increase of approximately 0.45 million parameters compared with SegUnet.

**Figure 6:**
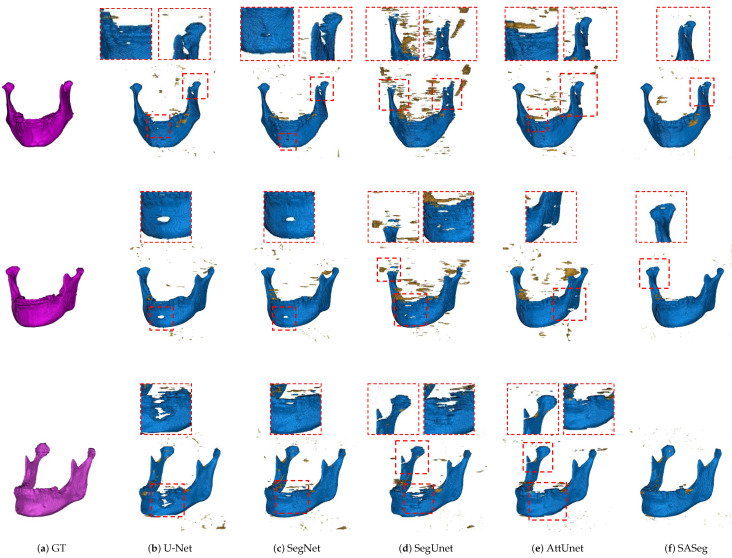
Results with the fashionable segmentation approaches. The ground truth is shown in the column (**a**). Columns (**b**–**f**) show the mandible predictions generated by the U-Net, SegNet, SegUnet, AttUnet, and the proposed SASeg, respectively. The red rectangle indicates the zoom-in views of bad predictions.

We also illustrate the 3D view of three examples taken from the dataset in Figure 6. Comparing the ground truth in Figure 6a, the 3D-based segmentation approaches shown in Figure 6 failed to segment the mandible detailed structures, such as the coronoids and parts of the mandible body indicated by the red rectangle. More concretely, the U-Net, SegNet, and SegUnet methods have a weaker ability to handle the mandible body that is close to the teeth and are affected by strong metal artifacts, and the segmentation results appear undersegmented, as shown in Figure 6b–d. Additionally, the SegUnet misclassifies the maxilla bone as the mandible, while the proposed SASeg can accurately segment the mandible. The AttUnet approach can address the mandible body but cannot correctly recognize the mandible angle, as illustrated in Figure 6e. As shown in Figure 6, these above-mentioned fashionable methods have problems with oversegmentation or undersegmentation while addressing the dental CBCT scans, while our method can precisely segment the entire mandible structure even if metal artifacts appear. Moreover, only a low number of voxels is misclassified by SASeg as the mandible. This can be explained by the fact that the proposed SASeg method aggregates pixelwise contextual information, resulting in better segmentation predictions and being able to learn spatial discrepancies between real mandible areas and other structures with high intensity in dental CBCT scans.

### 3.2. Ablation Experiments

Ablation tests are performed to analyze the influence of the components, i.e., the recurrent module and the PSFE module, and the loss function of the proposed SASeg approach. The final comparison of the experiments is also evaluated by calculating Dice, $D_{ASD}$ and $D_{95HD}$ between the ground truth and the automated segmentation.

#### 3.2.1. Ablation Analysis of the PSFE Module

To validate the effectiveness of our prior shape extractor module, we conduct a set of ablation experiments on the CBCT dataset. The experimental results are summarized in Table 2. From Table 2, we can see the use of PSFE and RNN modules brings the most gains in Dice, $D_{ASD}$, and $D_{95HD}$. When the network utilizes RNN modules, the continuous relationship between current slices is utilized and mined. In addition, the PSFE module is employed for further integrating refined contextual information after RNN modules from cross slices. From Table 2, by fully extracting the features of the mean mandible shape prior and integrating the information of different adjacent slices, our model achieves promising results.

**Figure 7:**
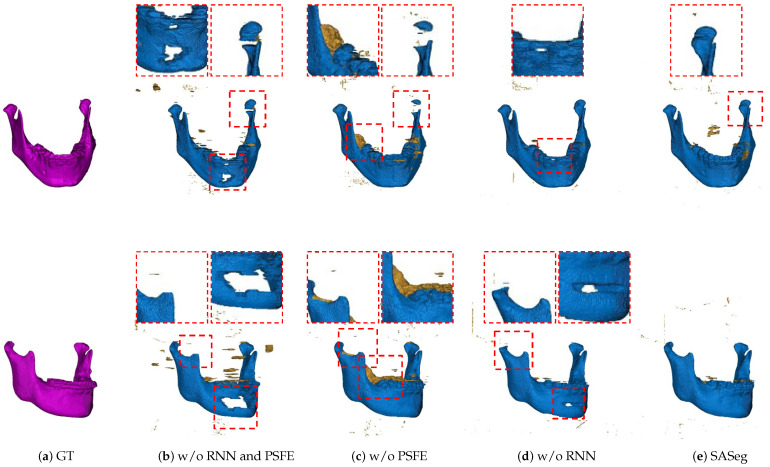
Results with different ablations of our method. The ground truth is shown in column (**a**). Columns (**b**–**e**) illustrate the segmentation results derived from the model without RNN and PSFE modules, without the PSFE module, and without the RNN module and the proposed SASeg, respectively. The red rectangle indicates the zoom-in views of bad predictions. (w/o RNN and PSFE: without RNN and PSFE modules, w/o PSFE: without the PSFE module, and w/o RNN: without the RNN module.)

We also show the effectiveness of the RNN and PSFE modules based on a mean shape model as a prior input by showing visualized results, as shown in Figure 7. We can obviously see that the method without the RNN and PSFE cannot address the mandible body affected by metal artifacts and condyles, as illustrated in Figure 7b. The method without PSFE easily causes oversegmentation in the angle of the mandible and is still not sensitive enough in the thick part such as condyles, as shown in Figure 7c. The method without the RNN in Figure 7d has a slightly stronger ability to handle the mandible body compared with the method without the RNN and PSFE. Incorporation of prior knowledge and spatial information into the mandible segmentation task could provide more accurate and reliable results in terms of the mandible anatomy.

#### 3.2.2. Ablation Analysis of the Loss Functions

**Figure 8:**
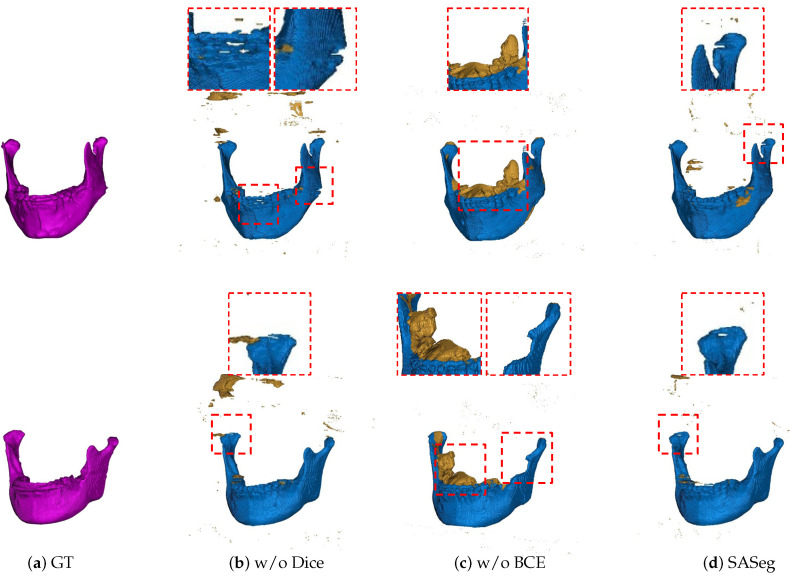
Results with different ablations of loss functions. The ground truth is shown in column (**a**). Columns (**b**–**d**) illustrate the segmentation results derived from the model without Dice loss, BCE loss and the proposed SASeg, respectively. The red rectangle indicates the zoom-in views of bad predictions. (w/o Dice: without Dice loss, w/o BCE: without BCE loss.)

In a similar way, we also perform a set of experiments on different loss settings based on the proposed method. The experimental results are listed in Table 3. As illustrated in Table 3, only BCE loss has a slightly higher performance than the combined loss of BCE and Dice in the Dice score, while the combined loss of BCE and Dice has higher performance in $D_{ASD}$ and $D_{95HD}$. It is worth noting that the number of predicted background pixels using BCE and Dice is more than the number of only using BCE, and the model using BCE and Dice is able to predict the fine appearance features of the mandible, as shown in Figure 8. In brief, the network with BCE and Dice as loss functions easily generates fewer false positives in mandible segmentation and is more focused on the structure of the object, as illustrated in Table 3 and Figure 8.

### 3.3. Reliability Analysis

To investigate the reliability of mandible segmentation, five randomly selected scans are used to evaluate the intraobserver variability and interobserver variability. For the intraobserver variability study, the second annotations are done six months after the first annotation, and for evaluating interobserver variability, we also employ another technical physician to annotate the mandible.

Intraobserver variability is the variability between the first and the second annotations of the first observer. The interobserver variability is the average variability between the second observer’s annotation and the first observer’s two annotations. Intra- and interobserver reliability tests for the mandible were computed using Dice, ASD, and 95HD. The intra- and interobserver agreements for manual segmentation are given in Table 4. For intraobserver variability, 98.76%, 0.0690 mm, and 0.6347 mm are found for mean Dice, $D_{ASD}$ and $D_{95HD}$, respectively. For interobserver variability, 91.56%, 0.3555 mm, and 2.0780 mm are obtained for mean Dice, $D_{ASD}$ and $D_{95HD}$, respectively, as shown in Table 4. It is worth noting that the Dice score for SASeg is higher than the interobserver reliability (95.35%>91.56%), and the $D_{95HD}$ value is slightly larger than interobserver reliability (2.5723>2.0780), demonstrating the reliability and robustness of the automatic segmentation with SASeg.

### 3.4. Experiments on the PDDCA Dataset

We evaluate the performance of the models on the test subset of PDDCA. As shown in Table 5, the proposed SASeg method achieves the best performance compared with the state-of-the-art methods, with the highest Dice score of 95.29%, the lowest $D_{ASD}$ of 0.1353 mm and the second lowest $D_{95HD}$ of 1.3054 mm.

## 4. Discussion

In this work, we developed and validated a novel deep learning-based approach (SASeg) for automated mandible segmentation that utilizes the PSFE module based on a mean mandible shape as a prior and a recurrent network to train the neural network model. In this way, the network makes segmentation predictions that are in agreement with the learned shape model of the underlying anatomy, which is referred to as a shape prior. Most importantly, the proposed approach allows us to perform fully 3D mandible segmentation via slice-by-slice 2D segmentation even in the presence of strong metal artifacts.

We first demonstrate the applicability of the proposed approach SASeg for a dental CBCT dataset of orthodontic treatment that is composed of 59 patient scans. Automated segmentation correlates well with manual segmentations, and the promising segmentation results are shown in Table 1, Table 2 and Table 3 and Figure 6, Figure 7 and Figure 8. As can be seen in the results, the existing state-of-the-art convolutional neural network (CNN) approaches for segmentation tasks perform poorly when the input data are strongly noisy and blurred by metal artifacts. The experiments described in Figure 6 and Table 1 show that the proposed segmentation models become more robust against CBCT imaging metal artifacts that are shown in Figure 1. The experimental results show that the classical network can benefit from the learned prior in cases where the images are corrupted by metal artifacts. Compared with the state-of-the-art methods that directly segment a single slice without considering the prior information and the continuity of neighborhood slices, the SASeg network provided better $D_{ASD}$ and $D_{95HD}$ scores. Figure 7 and Table 2 demonstrate that the mandible shape prior learned by the proposed shape extractor module (PSFE) is useful for mandible segmentation on dental CBCTs in orthodontics. As discussed in the above analysis, the added PSFE module that extracts the prior information from the mean mandible model plays an important role in the segmentation operation in that it can provide more focus on a certain region containing the mandible and avoid the influence of metal artifacts and other bone-structure organs. Figure 8 and Table 3 show that the combo loss function of Dice and BCE facilitate the network to focus more on the mandible and accordingly generate fewer false positives. Furthermore, we also investigate the intraobserver variability and interobserver variability for manual segmentation. Table 4 shows that a higher accuracy is achieved on mandible segmentation using the proposed SASeg when compared to interobserver variability. Furthermore, the PDDCA dataset, consisting of 40 patients scans, is employed for testing the proposed method. The quantitative results shown in Table 5 demonstrate that the proposed SASeg method has a good generalization ability in a conventional CT dataset. Overall, SASeg enables the algorithm to not only solve the challenge of mandible segmentation in a dental CBCT dataset with strong metal artifacts but also to provide a good approach to segment the mandible in a CT dataset.

There are a few limitations to the study. First, the CBCT dataset belonged to only a select patient group that required orthodontic treatment. The metal implants in orthognathic surgery should be included in the future. Second, the CBCT and PDDCA datasets are limited and cannot fully represent the general patient population in the clinical setting. Third, a mean mandible model is required before training the model. Fourth, we only use CBCT data from local institutes to train the SASeg model, and the PDDCA dataset in the external validation is a CT dataset and not a CBCT dataset.

To summarize, the proposed SASeg can anatomically and sequentially learn the 3D underlying anatomy through the auxiliary path and the recurrent module, respectively, which is able to enforce that network predictions follow the learned mean shape of the mandible structure and consider the continuity of neighborhood slices for the input scans. More importantly, it is easy to combine with any of the state-of-the-art medical image segmentation networks and can potentially improve their prediction accuracy and robustness with a slight increase in computational resources and memory. Last but not the least, training with the proposed mandible shape prior almost removes the need for postprocessing steps, which provides the capability of simplifying the postprocessing in segmentation tasks, especially in cases where the images are corrupted by metal artifacts or are noisy due to the limitations in their image acquisition. The accurate automated mandible segmentation offers an improved and faster procedure than clinical assessment in 3D VSP, thus providing more practical and faster therapy planning for surgeons or medical technician. The proposed SASeg model can be regarded as an application-specific training target. The presented SASeg framework is not limited to only the mandible segmentation task but can also be extended to other segmentation tasks in which prior knowledge can provide model guidance and robustness. In that regard, future research will focus on the application of SASeg to problems such as other anatomical organ segmentation even on low-quality scans.

## 5. Conclusions

In this paper, we propose an end-to-end approach (SASeg) for accurate mandible segmentation from CBCT scans that are badly influenced by metal artifacts. First, we adopt a PSFE module that encodes the shape’s prior information from a mean mandible model, and then we add it as an auxiliary path to assist the recurrent segmentation network to further learn the structure of the mandible. In this way, the proposed SASeg can automatically aggregate contextual information of the mandible at pixel level and capture the blurry mandible area without any interaction. The quantitative and qualitative results demonstrate that the proposed SASeg can yield better segmentation results compared to the other state-of-the-art algorithms.

## Figures and Tables

**Table 1:** Quantitative comparison of segmentation performance in the CBCT dataset between the proposed SASeg and the state-of-the-art methods.

Methods	Dice (%)	$D_{\mathrm{ASD}}$ (mm)	$D_{95\mathrm{HD}}$ (mm)	#Params (M)
U-Net	94.79 (±1.77)	2.0698 (±0.6137)	32.6401 (±22.0779)	3.35
SegNet	94.93 (±1.74 )	1.7762 (±1.5937)	15.9851 (±26.5286)	2.96
SegUnet	91.27 (±5.13)	3.1436 (±3.6049)	26.3569 (±34.9539)	3.35
AttUnet	93.34 (±3.79)	3.9705 (±4.6460)	35.1859 (±42.3474)	8.73
SASeg	95.35 (±1.54)	0.9908 (±0.4128)	2.5723 (±4.1192)	3.80

**Table 2:** Ablation analysis on different modules of the RNN and PSFE.

RNN	PSFE	Dice (%)	$D_{\mathrm{ASD}}$ (mm)	$D_{95\mathrm{HD}}$ (mm)
		93.78 (±2.50)	1.5851 (±1.0680)	17.2517 (±24.2681)
✓		92.26 (±5.66)	1.3133 (±0.7276)	7.2442 (±8.9275)
	✓	95.09 (±1.47)	1.2083 (±0.3354)	4.7629 (±8.1762)
✓	✓	95.35 (±1.54)	0.9908 (±0.4128)	2.5723 (±4.1192)

**Table 3:** Ablation analysis on different loss function settings.

BCE	Dice	Dice (%)	$D_{\mathrm{ASD}}$ (mm)	$D_{95\mathrm{HD}}$ (mm)
✓		95.45 (±1.39)	1.3934 (±0.6228)	10.6093 (±20.8654)
	✓	83.75 (±15.59)	3.2537 (±3.4075)	16.7939 (±23.4980)
✓	✓	95.35 (±1.54)	0.9908 (±0.4128)	2.5723 (±4.1192)

**Table 4:** Intra- and interobserver reliabilities and agreement of manual segmentation by the first expert.

	Dice (%)	$D_{\mathrm{ASD}}$ (mm)	$D_{95\mathrm{HD}}$ (mm)
Intraobserver	98.76 (±0.96)	0.0690 (±0.534)	0.6347 (±0.6176)
Interobserver	91.56 (±4.45)	0.3555 (±0.1701)	2.0780 (±1.1699)
SASeg	95.35 (±1.54)	0.9908 (±0.4128)	2.5723 (±4.1192)

**Table 5:** Quantitative comparison of the segmentation performance on the PDDCA dataset between the proposed method and the state-of-the-art methods.

Methods	Dice (%)	$D_{\mathrm{ASD}}$ (mm)	$D_{95\mathrm{HD}}$ (mm)
Multiatlas [49]	91.7 (±2.34)	-	2.4887 (±0.7610)
AAM [50]	92.67 (±1)	-	1.9767 (±0.5945)
ASM [51]	88.13 (±5.55)	-	2.832 (±1.1772)
CNN [52]	89.5 (±3.6)	-	-
NLGM [53]	93.08 (±2.36)	-	-
AnatomyNet [21]	92.51 (±2)	-	6.28 (±2.21)
FCNN [30]	92.07 (±1.15)	0.51 (±0.12)	2.01 (±0.83)
FCNN+SRM [30]	93.6 (±1.21)	0.371 (±0.11)	1. 5 (±0.32)
CNN+BD [54]	94.6 (±0.7)	0.29 (±0.03)	-
HVR [55]	94.4 (± 1.3)	0.43 (± 0.12)	-
Cascade 3D U-Net [56]	93 (±1.9)	-	1.26 (±0.5)
Multiplanar [12]	93.28 (±1.44)	-	1.4333 (±0.5564)
Multiview [57]	94.1 (±0.7)	0.28 (±0.14)	-
RSegUnet [26]	95.10 (±1.21)	0.1367 (±0.0382)	1.3560 (±0.4487)
SASeg	95.29 (±1.16)	0.1353 (±0.0481)	1.3054 (±0.3195)

## Data Availability

The Public Domain Database for Computational Anatomy Dataset (PDDCA) is available in https://www.imagenglab.com/newsite/pddca/ (accessed on 24 January 2019). Unfortunately, for reasons of ethics and patient confidentiality, we are not able to provide the sequencing data into a public database. The data underlying the results presented in the study are available from the corresponding author.
